# Neuroprotection by *Wld*^S^ depends on retinal ganglion cell type and age in glaucoma

**DOI:** 10.1186/s13024-021-00459-y

**Published:** 2021-06-05

**Authors:** Michael L. Risner, Silvia Pasini, Nolan R. McGrady, Karis B. D’Alessandro, Vincent Yao, Melissa L. Cooper, David J. Calkins

**Affiliations:** Department of Ophthalmology and Visual Sciences, Vanderbilt Eye Institute, Vanderbilt University Medical Center, AA7103 MCN/VUIIS, 1161 21st Ave. S, Nashville, TN 37232 USA

**Keywords:** Slow Wallerian degeneration, *Wld*^S^, Neuroprotection, Neurodegeneration, Glaucoma, Retinal ganglion cells, Axonopathy, Dendritic morphology

## Abstract

**Background:**

Early challenges to axonal physiology, active transport, and ultrastructure are endemic to age-related neurodegenerative disorders, including those affecting the optic nerve. Chief among these, glaucoma causes irreversible vision loss through sensitivity to intraocular pressure (IOP) that challenges retinal ganglion cell (RGC) axons, which comprise the optic nerve. Early RGC axonopathy includes distal to proximal progression that implicates a slow form of Wallerian degeneration. In multiple disease models, including inducible glaucoma, expression of the slow *Wallerian degeneration* (*Wld*^S^) allele slows axon degeneration and confers protection to cell bodies.

**Methods:**

Using an inducible model of glaucoma along with whole-cell patch clamp electrophysiology and morphological analysis, we tested if *Wld*^S^ also protects RGC light responses and dendrites and, if so, whether this protection depends upon RGC type. We induced glaucoma in young and aged mice to determine if neuroprotection by *Wld*^S^ on anterograde axonal transport and spatial contrast acuity depends on age.

**Results:**

We found *Wld*^S^ protects dendritic morphology and light-evoked responses of RGCs that signal light onset (αON-Sustained) during IOP elevation. However, IOP elevation significantly reduces dendritic complexity and light responses of RGCs that respond to light offset (αOFF-Sustained) regardless of *Wld*^S^. As expected, *Wld*^S^ preserves anterograde axon transport and spatial acuity in young adult mice, but its protection is significantly limited in aged mice.

**Conclusion:**

The efficacy of *Wld*^S^ in conferring protection to neurons and their axons varies by cell type and diminishes with age.

## Background

Age-related neurodegenerative disorders of the brain differ in etiology but often share similar important features [1, 2]. Among these are early challenges to axonal physiology, active transport, and ultrastructure [1, 3]. These pathological features are also endemic in diseases that affect the optic nerve, which conveys visual signals from the retina to central projection sites in the brain. Glaucomatous optic neuropathy (or glaucoma) is characterized by early axonopathy, including deficits in axonal anterograde transport from retina to central brain targets [4, 5]. The disease causes vision loss through progressive degeneration of retinal ganglion cells (RGCs) and their axons, which comprise the optic nerve. The disease remains a prevalent neurodegenerative disorder and is the leading cause of irreversible blindness worldwide, with an estimated 100 million people afflicted by 2040 [6]. Sensitivity to intraocular pressure (IOP) is an associated risk factor for glaucoma, and higher IOP is linked to accelerated pathology [5, 7, 8]. Thus, current treatments focus on lowering IOP. However, regardless of hypotensive therapeutic intervention, many patients continue to lose vision [9]. Thus, like age-related diseases of the brain itself, therapeutics are needed that address neurodegeneration directly [10].

Abating axon dysfunction early in neurodegeneration can prevent subsequent stages of glaucomatous progression [2, 3, 11, 12]. This is so as well in experimental models of glaucoma, which generally rely upon inducible or transgenic-related elevations in IOP [13, 14]. In rodents, experimental interventions that slow or prevent loss of anterograde transport to RGC projection sites in the brain also impede axon degeneration in the optic nerve and cell body loss in the retina [5, 15–17]. Without intact transport, axons undergo Wallerian or Wallerian-like degeneration [1]. Expression of the *Wld*^S^ (slow *Wallerian degeneration*) allele significantly delays axon degeneration in numerous disease models [18], though protection is dose- and possibly age-dependent [19, 20]. In glaucoma models, *Wld*^S^ preserves RGC axon structure, delays cell body loss, and preserves measures of gross retinal function [21–25]. The *Wld*^S^ mutation creates a chimeric fusion protein containing 70 N-terminal amino acids of ubiquitination factor *Ube4b* linked to full-length nicotinamide mononucleotide adenylyltransferase 1 or *Nmnat1*, the enzyme responsible for synthesis of nicotinamide adenine dinucleotide or NAD*,* a cofactor involved in oxidative phosphorylation [26–28]. Glaucoma compromises RGC axon metabolism long before outright degeneration primarily through elevated oxidative stress and reduced mitochondrial efficiency [4, 5, 29]. Supplementation with nicotinamide, a NAD precursor, preserves RGC metabolism, anterograde axon transport and optic nerve integrity in the DBA/2 J mouse model of glaucoma [24, 30].

Here we asked whether *Wld*^S^ also confers protection to RGC dendritic arbors in the retina and, if so, whether this protection depends upon RGC type. In glaucoma, RGCs that depolarize to light onset (ON cells) or light offset (OFF cells) may show different susceptibility to IOP elevations [31–33]. Using our microbead occlusion paradigm [14, 29, 34], we elevated IOP (+ 33%) in *Wld*^S^ mice and recorded light responses from a major class of ON and OFF RGCs, the α-Sustained type [32, 33, 35, 36]. By comparing wild-type (WT) and *Wld*^S^ RGC responses and morphology, we find that *Wld*^S^ protects αON-Sustained light responses and dendritic morphology following IOP elevation for 1 month. However, *Wld*^S^ αOFF-Sustained RGCs showed reduced light responses and dendritic complexity, similar to WT RGCs. Interestingly, while *Wld*^S^ preserved anterograde axonal transport to the brain and spatial contrast acuity in young mice, its protection diminished with age.

## Methods

### Animals

The Vanderbilt University Institutional Animal Care and Use Committee approved all experimental procedures described below. We purchased 6 to 8-week-old and 1-year old male wild type C57BL/6 mice (WT) from the Charles River Laboratory (Wilmington, MA). Two breeding pairs of homozygous slow Wallerian degeneration mutant mice (*Wld*^S^) on C57BL/6 background were kindly donated by Dr. Shu-Wei Sun from Loma Linda University [37]. We bred *Wld*^S^ mice in the Vanderbilt University Medical Center Division of Animal Care. For our experiments we used 6 to 10-week-old and 1-year old male and female homozygous *Wld*^S^ mice (see Genotype Confirmation). Mice were maintained on a 12-h light-dark cycle with standard rodent chow available ad libitum.

### Genotype confirmation

**Fig. 1:**
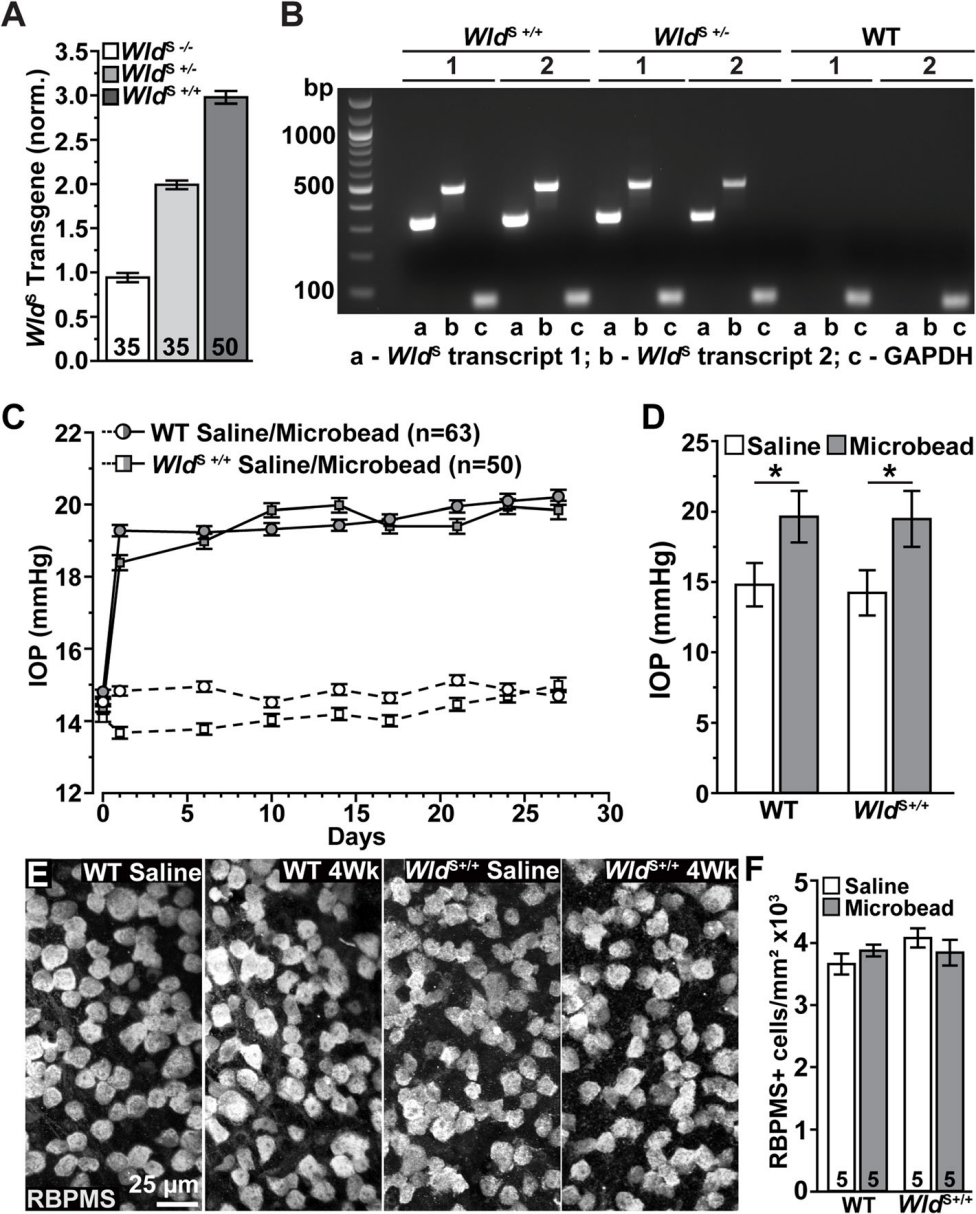
Genotype Confirmation, IOP Elevation, and RGC Density Measurements. **A** Quantitative RT-PCR measurements of *Wld*^S^ transgene in genomic DNA from *Wld*^S^ null (*Wld*^S*−/−*^), *Wld*^S^ heterozygous (*Wld*^S*+/−*^), and *Wld*^S^ homozygous (*Wld*^S*+/+*^) mice. Values are normalized against WT animals. **B** Agarose gel (2%) showing RT-PCR detection in the retina of *Wld*^S^ chimeric (**a,b**) and housekeeping GAPDH (c) transcripts from *Wld*^S*+/+*^, *Wld*^S*+/−*^, and WT mice. 1,2 indicates samples extracted from different mice. **C** Mean intraocular pressure (IOP) in WT (circles) and *Wld*^S*+/+*^ (squares) mice before (day 0) and following (days ≥1) a single unilateral injection of polystyrene microbeads (gray symbols) or equivalent volume of saline (white symbols). **D** Microbead injection significantly elevated IOP in all genotypes (WT: 19.64 ± 1.82 vs. 14.81 ± 1.54 mmHg, *p* < 0.001; *Wld*^S*+/+*^: 19.48 ± 1.98 vs. 14.23 ± 1.61 mmHg, *p* < 0.001). **E** Representative confocal images of whole-mount retinas from WT and *Wld*^S*+/+*^ saline and microbead injected eyes immunolabeled against RBPMS (grayscale). Scale bar = 25 μm. **F** When quantified, we did not detect a significant difference in RBPMS+ RGCs regardless of genotype or IOP condition (*p* = 0.389). Statistics: Student’s *t*-test (**D**). One-Way ANOVA Tukey’s Post hoc (**F**). Data = mean ± SEM

We genotyped *Wld*^S^ mice following the published protocol [38]. Briefly, we extracted genomic DNA from tails (DNeasy Blood & Tissue, Qiagen, Hilden, Germany). Because the *Wld*^S^ mutation contains an in tandem triplication of a region intrinsic to the mouse genome, we determined the copy number of mutant *Wld*^S^ alleles (QuantStudio 3 Real Time PCR, Applied Biosystems, Waltham, MA) using the following primers: *Wld*^S^ forward: 5′-GGC AGT GAC GCT CAG AAA TTC-3′ and *Wld*^S^ reverse: 5′- GTT CAC CAG GTG GAT GTT GCT-3′. β-tubulin forward: 5′-GCC AGA GTG GTG CAG GAA ATA-3′ β-tubulin reverse: 5′-TCA CCA CGT CCA GGA CAG AGT-3′. For each mouse, *Wld*^S^ allele copy number was measured in triplicate, normalized to β-tubulin as endogenous control, and we quantified the copy number using the comparative ΔCT method [39]. We then normalized *Wld*^S^ copy number relative to *Wld*^S^ copy number measured in WT C57 mice obtained from Charles River Laboratory. As previously observed [38], *Wld*^S^ copies formed a continuum. We defined animal genotype by *Wld*^S^ copies using criteria established by [38]. *Wld*^S^ null mice copy number ranged from 0 to 1.4 (*Wld*^S−/−^), *Wld*^S^ heterozygotes 1.4–2.5 (*Wld*^S+/−^), and *Wld*^S^ homozygotes ≥2.5 (*Wld*^S+/+^, Fig. 1A).

We confirmed the expression of *Wld*^S^ transcripts in retinas of suspect *Wld*^S*+/+*^, *Wld*^S*+/−*^, and C57 WT mice following the protocol described by [27]. Briefly, we euthanized mice by cervical dislocation and quickly removed retinas. We then extracted total RNA based on manufacturer’s protocol (SV Total RNA Isolation System, Promega, Madison, WI). Next, we determined RNA concentration and purity (NanoDrop 8000, Thermo Scientific, Wilmington, DE). Afterward, we reverse-transcribed mRNA into cDNA (1st Strand cDNA Synthesis System, Origene, Rockville, MD) and amplified the cDNA (QuantStudio 3 Real Time PCR System, Applied Biosystems, Waltham, MA) using the PowerUp SYBR Green Master Mix (Applied Biosystem, Waltham, MA) and the following primers: *Wld*^S^ transcript 1: forward (PE9): 5′-CAC GAC TTG CTG GTG GAC AGA-3′ and reverse (PE4) 5′-GTC CTTGGC CAG CTC GAA CA-3′; *Wld*^S^ transcript 2: forward (PE9): 5′ CAC GAC TTG CTG GTG GAC AGA-3′ and reverse (PE6) 5′-TTT CCC ACG TAT CCA CTT CCA-3′. We used GAPDH as housekeeping gene; forward: 5′-TCC ATG ACA ACT TTG GCA TTG-3′ and reverse 5′-CAG TCT TCT GGG TGG CAG TGA-3′. *Wld*^S^ transcripts were distinguished by electrophoresis of the RT-PCR product (2% agarose, 2% EtBr) and confirmed (ChemiDoc MP Imaging System, Bio-Rad, Hercules, CA, Fig. 1B).

### Intraocular pressure elevation

Prior to intraocular pressure (IOP) elevation, baseline IOP was measured on 2 separate days by rebound tonometry (Tono-Pen XL; Medtronic Solan, Waltham MA) in anesthetized (2.5% isoflurane) mice. IOP was elevated in anesthetized (2.5% isoflurane) mice by a single unilateral injection of 1.5 μL polystyrene microbeads (15 μm, Invitrogen, Carlsbad CA) into the anterior chamber. We injected sterile phosphate buffered saline (PBS) into the contralateral eye to serve as an internal control. We measured IOP in anesthetized (2.5% isoflurane) mice bi-weekly [14, 29, 40–43]. For all mice used in this study, a single microbead injection increased IOP ~ 30% compared to saline-injected eyes, and IOP remained elevated for the duration (4 weeks, Fig. 1C, D).

### Retinal ganglion cell physiology

Mice were euthanized by cervical dislocation, and whole retinas were removed under long-wavelength illumination (630 nm, 800 μW/cm^2^, FND/FG, Ushio, Cypress, CA). To aid in the removal of vitreous membrane from the retinal surface, we incubated retinas in a solution containing collagenase (LS005273, Worthington Biochemical, Lakewood, NJ), hyaluronidase (LS002592, Worthington Biochemical, Lakewood, NJ), and carbogen-saturated Ames’ medium (US Biologic, Memphis, TN) for 10 mins on a rocker as described by [44]. Afterwards, retinas were rinsed within fresh Ames’ medium and placed into physiological chamber mounted on an upright microscope (BX50, Olympus, Center Valley, PA). Retinas were maintained in the dark at 32 °C (TC-344B; Warner Instruments), and constantly perfused (2 mL/min) with carbogen-saturated NaHCO_3_-buffered (22.6 mM) Ames’ medium plus 20 mM glucose (Osm 290, pH 7.4). For RGC intracellular filling and patch-clamp recordings, we used fire-polished borosilicate glass pipettes containing (in mM) 125 K-gluconate,10 KCl, 10 HEPES, 10 EGTA, 4 Mg-ATP, 1 Na-GTP, and 0.1 ALEXA 555 (Invitrogen, Carlsbad CA; Osm 285, pH 7.35). RGC light responses were evoked using full-field light flashes generated by a light-emitting diode (365 nm, 300 μW/cm2, 3-s duration; Roithner Lasertechnik) delivered through a shutter in the microscope condenser [29, 34].

### Retinal immunohistochemistry and imaging

After electrophysiological recordings, we fixed retinas overnight in 4% paraformaldehyde and immunolabeled them with the following primary antibodies: non-phosphorylated neurofilament H (SMI-32,1:1000; BioLegend, San Diego, CA), choline acetyltransferase (ChAT, AB144P,1:100, Millipore, Burlington, MA), and s-opsin (abn1660,1:500, Millipore). In a subset of experiments, we immunolabeled whole-mount retinas against RNA-binding protein with multiple splicing (RBPMS, GTX118619, GeneTex, Irving, CA). Retinas were incubated with appropriate secondary antibodies (1:200; Jackson ImmunoResearch Laboratories, Inc., West Grove, PA) and mounted with Fluoromount G (Southern Biotech, Birmingham, AL). We imaged retinas using an Olympus FV-1000 inverted confocal microscope. We measured the density of RBPMS-positive RGCs by capturing four 0.0448 mm^2^ images from whole-mounted retinas along the midline of each retinal quadrant. After imaging, we counted the number of cells positive for RBPMS, and we calculated RGC density as RGCs/mm^2^.

### Retinal ganglion cell dendritic morphology analysis

After imaging, micrographs of RGCs dendritic arbors were montaged and manually traced using Adobe Illustrator and Adobe Photoshop, respectively. We analyzed the skeletonized arbors by measuring the following parameters: dendritic field area, total dendritic length, dendritic intersections, and branch points. Dendritic field area was defined by outlining the distal dendritic tips and calculating the area of the resultant polygon. Total dendritic length was defined as the summation of all dendrites. The number of dendritic intersections was determined by Sholl analysis (ImageJ, 1.53c). Dendritic branch points were analyzed manually by counting the number of dendritic bifurcations [34].

### Anterograde axonal transport

We anesthetized mice with 2.5% isoflurane and injected 1.5 μL of 1 μg/ μL solution of cholera toxin subunit B (CTB) conjugated to Alexa Fluor 488 (Molecular Probes, Eugene, OR) into the vitreous of both eyes. Forty-eight hours later we perfused mice transcardially with PBS followed by 4% paraformaldehyde. Brains were removed and cryoprotected in 20% sucrose and coronal midbrain sections (50 μm) cut on a freezing sliding microtome. We imaged alternating sections of the superior colliculus (SC) using a Nikon Ti Eclipse microscope (Nikon Instruments Inc., Melville, NY) and quantified the intensity of CTB signal (intact transport) using a custom ImagePro macro (Media Cybernetics, Bethesda, MD) as previously described [4]. We confirmed CTB uptake by RGCs in the retinas using an Olympus FV-1000 inverted confocal microscope.

### Behavioral spatial acuity

We placed unrestrained mice on an elevated platform located in the center of an arena surrounded by four adjoining computer monitors (OptoMotry; Cerebral Mechanics Inc., Canada [45]). We measured spatial frequency thresholds by assessing the optomotor response to drifting sinusoidal gratings at 100% contrast. Grating spatial frequency was systemically adjusted based on the optomotor response noted by naïve experimenters. Mice were tested 3 times before microbead injection (baseline) and twice a week for 4 weeks after injection [29, 43]. We analyzed spatial acuity data by calculating the difference in spatial acuity (Δ spatial acuity) of microbead and saline injected eyes.

### Statistical analysis

All data are presented as mean ± standard error of the mean (SEM). Statistics were performed using Graphpad 8.0 (Graphpad Software, San Diego, CA). We first assessed data for outliers using Grubb’s test. Then, we determined whether data best fit normal or lognormal distributions. We used the Shapiro-Wilk test for normality. If data best fit a lognormal distribution, datasets were transformed by computing the logarithm (base 10) [34, 46]. For normal or transformed data, we performed parametric statistics. If datasets fit undetermined distributions or mix distributions (normal and lognormal), we performed non-parametric statistics. Statistical significance was defined as *p* ≤ 0.05.

## Results

### Neuroprotection by *Wld*^S^ is dependent on RGC type during glaucoma

We verified the genotype of each *Wld*^S^ mouse by measuring the number of transgene copies relative to C57Bl/6 (WT) mice. These formed a continuum ranging from null (*Wld*^S−/−^, corresponding to WT) to 2.5 and above (Fig. 1A), which we defined as *Wld*^S+/+^ following previous studies [27, 38]. For a subset of mice, we compared *Wld*^S*+/+*^, *Wld*^S+/−^, and WT designation in whole retina by probing for both *Wld*^S^ transcripts (318 bp and 478 bp) compared to GAPDH (71 bp; Fig. 1B). While naïve intraocular pressure (IOP) did not differ between WT left and right eyes nor between *Wld*^S+/+^ left and right eyes (*p* ≥ 0.17), we detected a modest difference in IOP between WT and *Wld*^S+/+^ naïve eyes (14.64 ± 0.13 vs. 14.1 ± 0.13 mmHg, respectively, *p* = 0.006). This small difference persisted in eyes receiving a saline injection as internal control (Fig. 1C) but was not significant when averaged over the one-month experimental period (*p* = 0.79, Fig. 1D). In contrast, an injection of microbeads into the anterior chamber following our established protocol elevated IOP in both WT (+ 32.6%, 19.64 ± 1.82 vs. 14.81 ± 1.5 mmHg) and *Wld*^S+/+^ (+ 36.8%, 19.48 ± 1.97 vs. 14.23 ± 1.61 mmHg) compared to saline-injected eyes (*p* < 0.001, Fig. 1D). Next, we determined if genotype or IOP elevation influenced RGC density by immunolabeling whole-mount retinas against RNA-binding protein with multiple splicing (RBPMS, [47], Fig. 1E). When quantified, we did not detect a significant difference in RBPMS+ RGCs regardless of genotype or IOP condition (WT saline: 3662.15 ± 142.12, WT microbead 3879.73 ± 155.85, *Wld*^S+/+^ saline: 4083.55 ± 154.86, *Wld*^S+/+^ microbead: 3845.82 ± 207.12 RBPMS+ RGCs/mm^2^, *p* = 0.389, Fig. 1F).

**Fig. 2:**
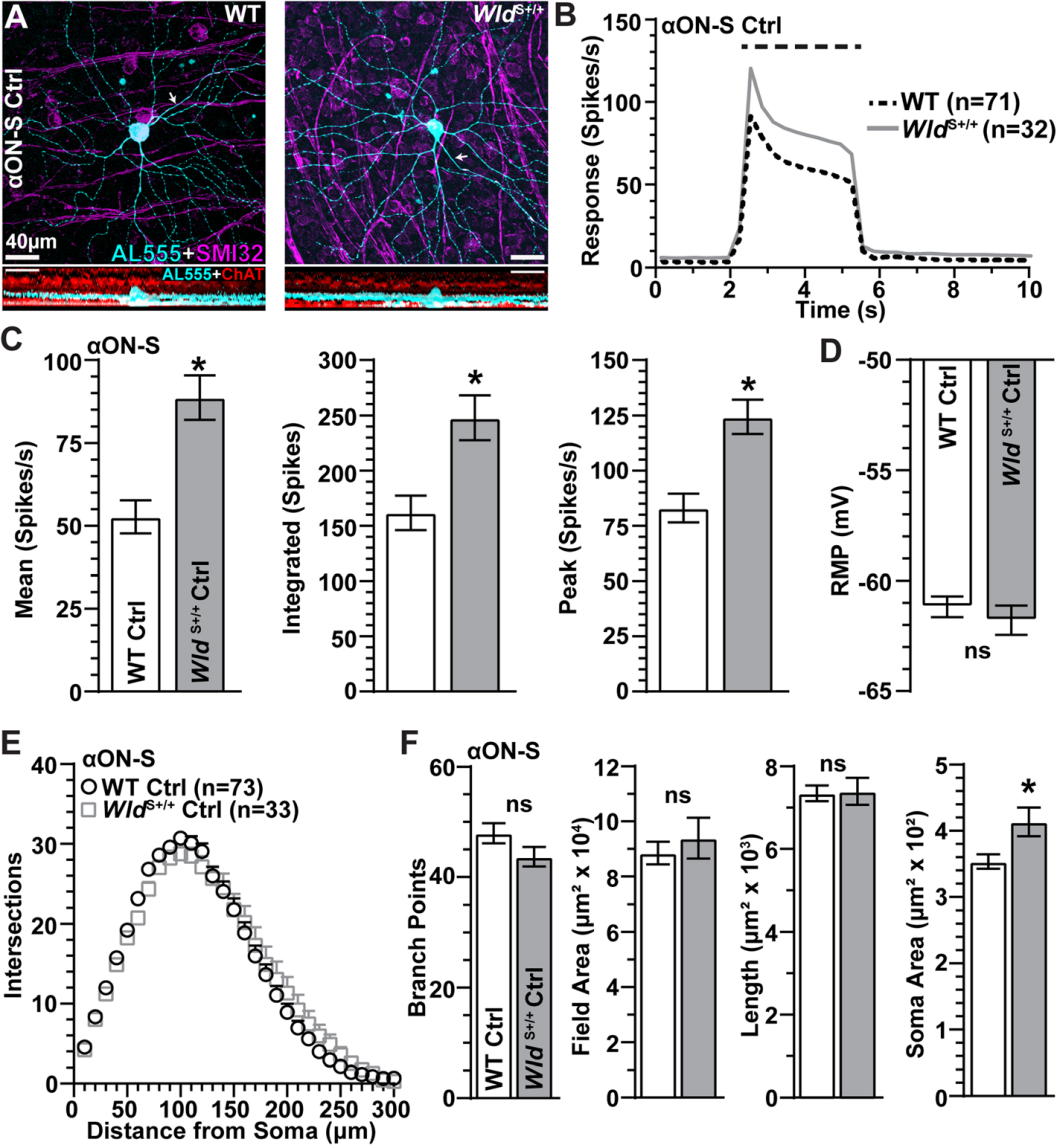
Morphological and Physiological Characterization of αON-Sustained RGCs in WT and *Wld*^S+/+^ Control Retinas. **A** Example *en face* (top) and orthogonal (bottom) images of dye-filled (AL555, cyan) αON-S RGCs from WT and *Wld*^S+/+^ Ctrl animals. αON-S RGCs were identified by positive immunolabeling against SMI-32 (magenta) and dendritic projections within the ON sublamina of the IPL identified by ChAT (red) immunolabeling. **B** Average light-evoked (3 s 365 nm, horizontal dashed line) spike rate histograms (300 ms bin width) of αON-S RGCs from WT and *Wld*^S+/+^Ctrl eyes. **C** *Wld*^S+/+^ Ctrl αON-S RGC light responses are more robust than WTs (*, *p* ≤ 0.008), **D** but RMP of WT and *Wld*^S+/+^ Ctrl αON-S RGCs is similar (*p* = 0.47). **E** Sholl analysis of WT and *Wld*^S+/+^ Ctrl αON-S RGCs. **F** Dendritic branch points, field area, and length are similar between WT and *Wld*^S+/+^ Ctrl αON-S RGCs (*p* ≥ 0.23), but *Wld*^S+/+^ somas are significantly larger (*p* = 0.01). WT Ctrl group contained cells from WT saline (*n* = 59) and WT naïve (*n* = 14) eyes. *Wld*^S+/+^ Ctrl group consisted of cells from *Wld*^S+/+^ saline-injected (*n* = 18) and naïve (*n* = 15) eyes. Statistics: Mann-Whitney tests ( **C**, **D**, **F**, Field Area, Soma Size); Two-Way Repeated Measures ANOVA, Bonferroni post hoc tests (**E**), Student’s *t*-tests (**F**, branch points and length). Scale bars = 40 μm. Arrows indicate RGC axons. Data = mean ± SEM

We targeted α-Sustained RGCs of the same type between WT and *Wld*^S+/+^ retinas based on large cell body, dendritic stratification within the inner plexiform layer (IPL), immunoreactivity for non-phosphorylated neurofilament H (SMI-32), and response to light following previous work [29, 32–34, 48]. Both WT and *Wld*^S+/+^ αON-sustained (αON-S) RGCs expressed SMI-32, with dendrites ramifying proximal to choline acetyltransferase (ChAT)-positive amacrine cell processes defining the “ON” sublamina of the IPL (Fig. 2A). These cells maintained a low spontaneous firing rate in darkness and produced a robust sustained train of action potentials in response to light, which appeared more robust for *Wld*^S+/+^ αON-S RGCs (Fig. 2B). When quantified, we found *Wld*^S+/+^ αON-S RGCs produced significantly greater responses to light as measured by the mean response (+ 68%, *p* < 0.001), the integrated response (+ 53%, *p* = 0.008), and the peak response to light compared to WT counterparts (+ 50%, *p* < 0.001; Fig. 2C). Even so, the resting membrane potential (RMP) of WT and *Wld*^S+/+^ control αON-S RGCs was similar (*p* = 0.47, Fig. 2D), suggesting these differences were not due to a higher degree of resting depolarization. When quantified by Sholl analysis, the dendritic morphology of WT and *Wld*^S+/+^ control αON-S RGCs were similar (*p* ≥ 0.52) as were the number of branch points (*p* = 0.23), field area (*p* = 0.79), and total dendrite length (*p* = 0.89; Fig. 2E, F). However, αON-S RGC somas from *Wld*^S+/+^ control retinas were significantly larger than WT counterparts (+ 17%, *p* = 0.01, Fig. 2F).

**Fig. 3:**
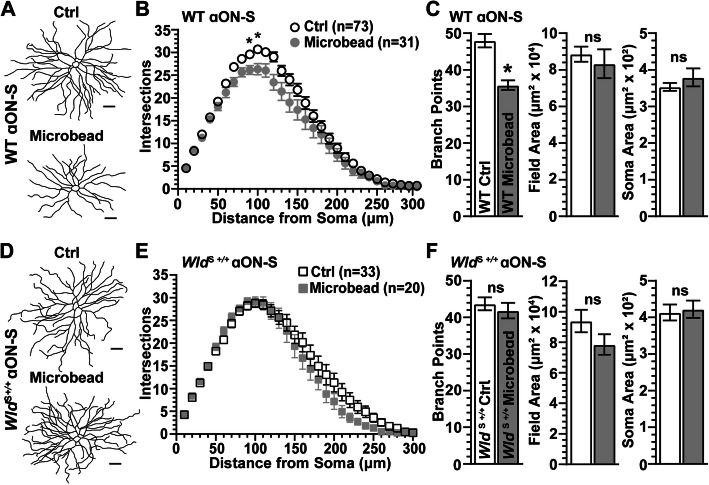
*Wld*^S*+/+*^ Prevents Dendritic Pruning of αON-S RGCs During IOP Elevation. **A** Reconstructed dendritic arbors of WT αON-S RGCs from Ctrl and microbead-injected eyes. **B** Sholl analysis shows IOP elevation reduced the number of dendritic intersections between 90 and 100 μm from soma (*p* ≤ 0.05) of WT αON-S RGCs. **C** Similarly, IOP elevation significantly decreased the number of branching points in WT αON-S RGC dendrites (*p* < 0.001). For WT αON-S RGCs, IOP elevation did not significantly affect dendritic field area (*p* = 0.14) or soma area (*p* = 0.25). **D** Reconstructed dendritic arbors of *Wld*^S+/+^ αON-S RGCs from Ctrl and microbead-injected eyes. **E** Sholl analysis shows IOP elevation does not significantly affect the number of dendritic crossings in *Wld*^S+/+^ αON-S RGC (*p* ≥ 0.37). **F** Dendritic branching (*p* = 0.51), field area (*p* = 0.30), and soma area (*p* = 0.79) of *Wld*^S+/+^ αON-S RGCs remain intact during IOP elevation. Statistics: Two-way repeated measures ANOVA, Bonferroni post hoc (**B, E**); Student’s *t*-test (**C, F**). Scale bars = 50 μm. Data presented as mean ± SEM

Previously we found that 1 month of IOP elevation reduces both dendritic complexity and RGC light responses, while 2 weeks enhances RGC excitability [29, 34]. Consistent with these results, 1 month of IOP elevation significantly decreased dendritic complexity of WT αON-S cells as measured by Sholl analysis (*p* ≤ 0.05, Fig. 3A, B). The number of dendritic branch points also significantly decreased (− 24%, *p* < 0.001), while dendritic field area and cross-sectional soma area did not change (*p* ≥ 0.14, Fig. 3C). In contrast, elevated IOP had little discernable effect on the complexity of αON-S RGCs from *Wld*^S+/+^ retina compared to control, again as measured by Sholl analysis (*p* ≥ 0.37; Fig. 3D, E). Similarly, *Wld*^S+/+^ protected dendritic branch points (*p* = 0.51), field area (*p* = 0.30), and soma area (*p* = 0.79, Fig. 3F).

**Fig. 4:**
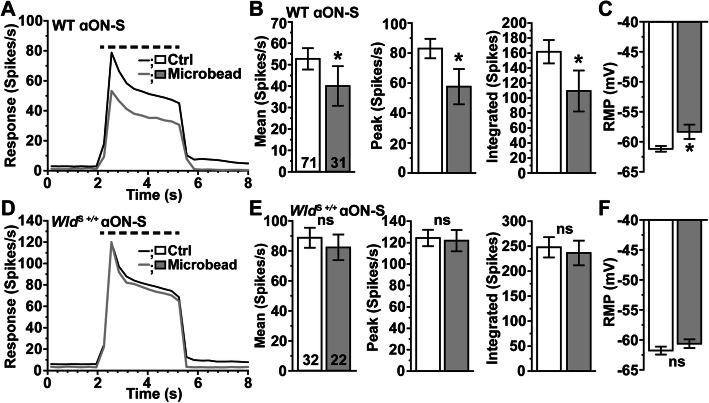
*Wld*^S+/+^ Protects αON-S RGC Light Responses and RMP During Glaucoma. **A** Mean light-evoked spike rate histograms (300 ms bin width) of αON-S RGCs from Ctrl and microbead eyes of WT mice. **B** IOP elevation reduced light-evoked average spike rate (− 23% 40.8 ± 9 vs. 52.7 ± 5 spikes/s, *p* = 0.002), peak spike rate (− 30%, 58.3 ± 11.5 vs. 83.1 ± 6.5 spikes/s, *p* = 0.0003), integrated response (− 31%, 111.7 ± 26.5 vs. 162 ± 15.6 spikes, *p* = 0.0001), and **C** depolarized RMP (− 58.3 ± 1.2 vs. -61.2 ± 0.5 mV, *p* = 0.02). **D** Mean light-driven spike rate histograms of αON-S RGCs from *Wld*^S+/+^ Ctrl and microbead eyes. **E** IOP elevation does not affect light-evoked mean firing rate (*p* = 0.55), peak spike rate (*p* = 0.84), integrated response (*p* = 0.72), or **F** RMP (*p* = 0.24) of *Wld*^S+/+^ αON-S RGCs. Statistics: Mann-Whitney test (**B, C, F**); Student’s *t*-test **(E)**. Data = mean ± SEM

As before [29], a month of elevated IOP reduced the response of αON-S RGCs from WT control retinas (Fig. 4A), including the mean (− 23%), peak (− 30%), and integrated (− 31%) response to light (*p* ≤ 0.002, Fig. 4B). The RMP was more depolarized (+ 5%) as well (*p* = 0.001, Fig. 4C). In contrast IOP did not influence the response of *Wld*^S+/+^ αON-S RGCs (Fig. 4D), as the mean, peak, and integrated response did not change relative to control (*p* ≥ 0.55, Fig. 4E). Unlike WT αON-S RGCs, RMP did not change with IOP elevation (*p* = 0.24, Fig. 4F). Thus, *Wld*^S+/+^ protects αON-S RGCs against stress-induced pruning of dendritic arbors and reductions in response that would ensue.

**Fig. 5:**
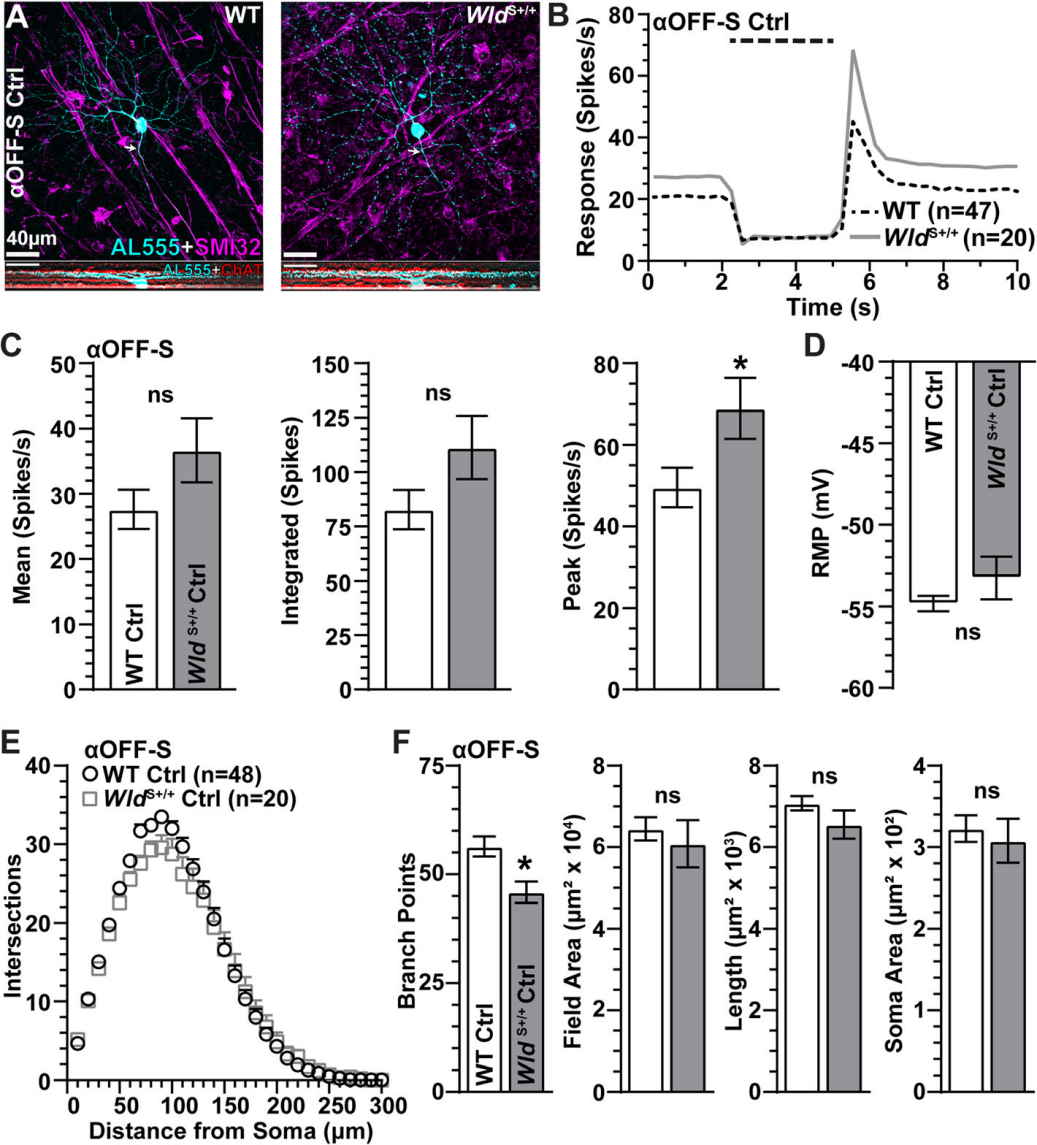
Morphological and Physiological Comparison of αOFF-Sustained RGCs from WT and *Wld*^S+/+^ Control Retinas. **A** Representative forward (top) and orthogonal (bottom) projection micrographs of dye-filled (AL555, cyan) αOFF-S RGCs from Ctrl eyes of WT and *Wld*^S+/+^ animals. αOFF-S RGCs were identified by modest immunolabeling against SMI-32 (magenta) and dendritic projections within the distal OFF sublamina of the IPL identified by ChAT (red) immunoreactivity. **B** Mean light-driven spike rate histograms of αOFF-S RGCs from WT and *Wld*^S+/+^ retinas from Ctrl eyes. **C** αOFF-S RGC light-evoked mean (*p* = 0.08), integrated (*p* = 0.07), and peak (*p* = 0.03) responses are enhanced by *Wld*^S+/+^ versus WT Ctrl cells. **D** RMP is similar for WT and *Wld*^*S*+/+^ Ctrl αOFF-S RGCs (*p* = 0.17). **E** Sholl analysis of WT and *Wld*^S+/+^ Ctrl αOFF-S RGC dendrites (*p* ≥ 0.09). **F** The average number of dendritic branch points of αOFF-S RGCs is reduced by *Wld*^S+/+^ (*p* = 0.005), but dendritic field area (*p* = 0.52), dendritic length (*p* = 0.14), and soma area (*p* = 0.50) are similar to WTs. WT Ctrl group consisted of cells from WT saline (*n* = 38) and WT naïve (*n* = 10) eyes. *Wld*^S+/+^ Ctrl group contained cells from *Wld*^S+/+^ saline-injected (*n* = 12) and naïve (*n* = 8) eyes. Statistics: Mann-Whitney test (**C**); Student’s *t*-test (**D**, **F**); Two-Way Repeated Measures ANOVA, Bonferroni post hoc tests (**E**). Scale bars = 40 μm. Data are expressed as mean ± SEM

Next, we examined the influence of *Wld*^S+/+^ on the morphology and physiology of αOFF-S RGCs with IOP elevation. Following previous work, we identified αOFF-S RGCs in WT and *Wld*^S+/+^ retinas based on dendritic projections within the IPL, immunoreactivity to SMI-32, and their responses to light [29, 32–34]. In WT and *Wld*^S+/+^ retinas, αOFF-S RGCs modestly express SMI-32 and have large somas with large, complex dendritic fields that project within the distal OFF sublamina of the IPL near ChAT-positive amacrine cell processes (Fig. 5A). These RGCs produced a high rate of spontaneous activity in darkness, which was inhibited by light onset, and generated a sustained train of action potentials following light offset (Fig. 5B). Like *Wld*^S+/+^ αON-S RGCs (Fig. 2B), the analogous response of *Wld*^S+/+^ αOFF-S RGCs was more robust than WT (Fig. 5B), with a significantly higher peak response (+ 28%, *p* = 0.03; Fig. 5C). As with αON-S RGCs, *Wld*^S+/+^ did not affect the RMP of αOFF-S cells (*p* = 0.17, Fig. 5D). Unlike αON-S RGCs, *Wld*^S+/+^ moderately reduced dendritic complexity of αOFF-S RGCs, with significantly less dendritic branch points (− 19%, *p* = 0.005, Fig. 5E, F). However, dendritic field area, total dendrite length, and soma area did not differ compared to WT (*p* ≥ 0.14, Fig. 5F).

**Fig. 6:**
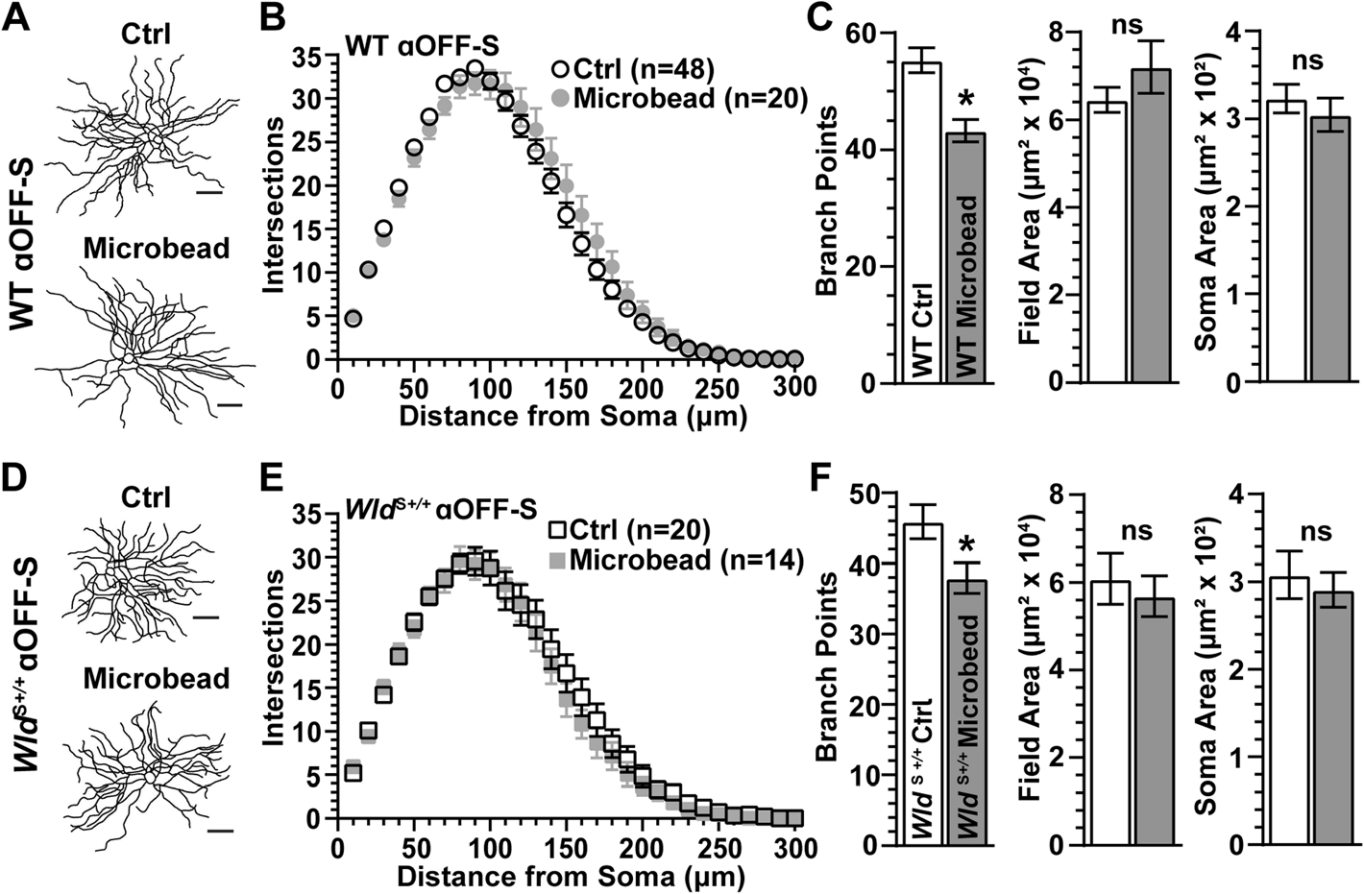
*Wld*^S+/+^ Does Not Prevent IOP-Induced Dendritic Degeneration in αOFF-S RGCs **A** Example tracings of WT αOFF-S RGC dendritic arbors from Ctrl and microbead-injected eyes. **B** Sholl analysis shows the number of dendritic crossings of WT αOFF-S RGC is unaffected by IOP elevation (*p* ≥ 0.79). **C** IOP elevation significantly reduced the number of dendritic branching points in WT αOFF-S RGC (43 ± 1.9 vs. 56.4 ± 2.3 branch points, *p* = 0.003), but dendritic field area (*p* = 0.20) and soma area (*p* = 0.63) remain intact. **D** Representative reconstructions of *Wld*^S+/+^ αOFF-S RGC dendritic arbors from Ctrl and microbead-injected eyes. **E** IOP elevation does not affect the number of dendritic intersections in *Wld*^S+/+^ αOFF-S RGCs (*p* > 0.99). **F** IOP elevation reduced the number of dendritic branching points in *Wld*^S+/+^ αOFF-S RGCs (38 ± 2.2 vs. 46 ± 2.4 branch points, *p* = 0.03), but elevating IOP did not affect dendritic field area (*p* = 0.62) or soma area (*p* = 0.90). Statistics: Two-Way Repeated Measures ANOVA, Bonferroni post hoc **(B, E)**; Student’s *t*-test **(C, F)**; Mann-Whitney test (**F**, soma area). Scale bars = 50 μm. Data = mean ± SEM

Based on Sholl analysis, a month of IOP elevation did not influence the number of dendritic intersections of WT αOFF-S RGCs (*p* ≥ 0.79, Fig. 6B). Even so, the number of branch points in these cells reduced by 23% compared to control cells (*p* = 0.003), even as dendritic field area and cross-sectional soma area did not change (*p* ≥ 0.20, Fig. 6C). Similarly, IOP elevation did not appear to alter dendritic morphology of *Wld*^S+/+^ αOFF-S RGCs (Fig. 6D). Based on Sholl analysis, IOP elevation did not significantly affect the number of dendritic crossings (*p* > 0.99). However, as with WT, *Wld*^S+/+^ αOFF-S RGCs demonstrated reduced branch points (− 17%, *p* = 0.03), but no change in dendritic field area or soma area (*p* ≥ 0.62; Fig. 6E, F). Thus, unlike αON-S RGCs (Fig. 3D-F), *Wld*^S+/+^ does not prevent loss of dendritic branch points in αOFF-S RGCs following IOP elevation.

**Fig. 7:**
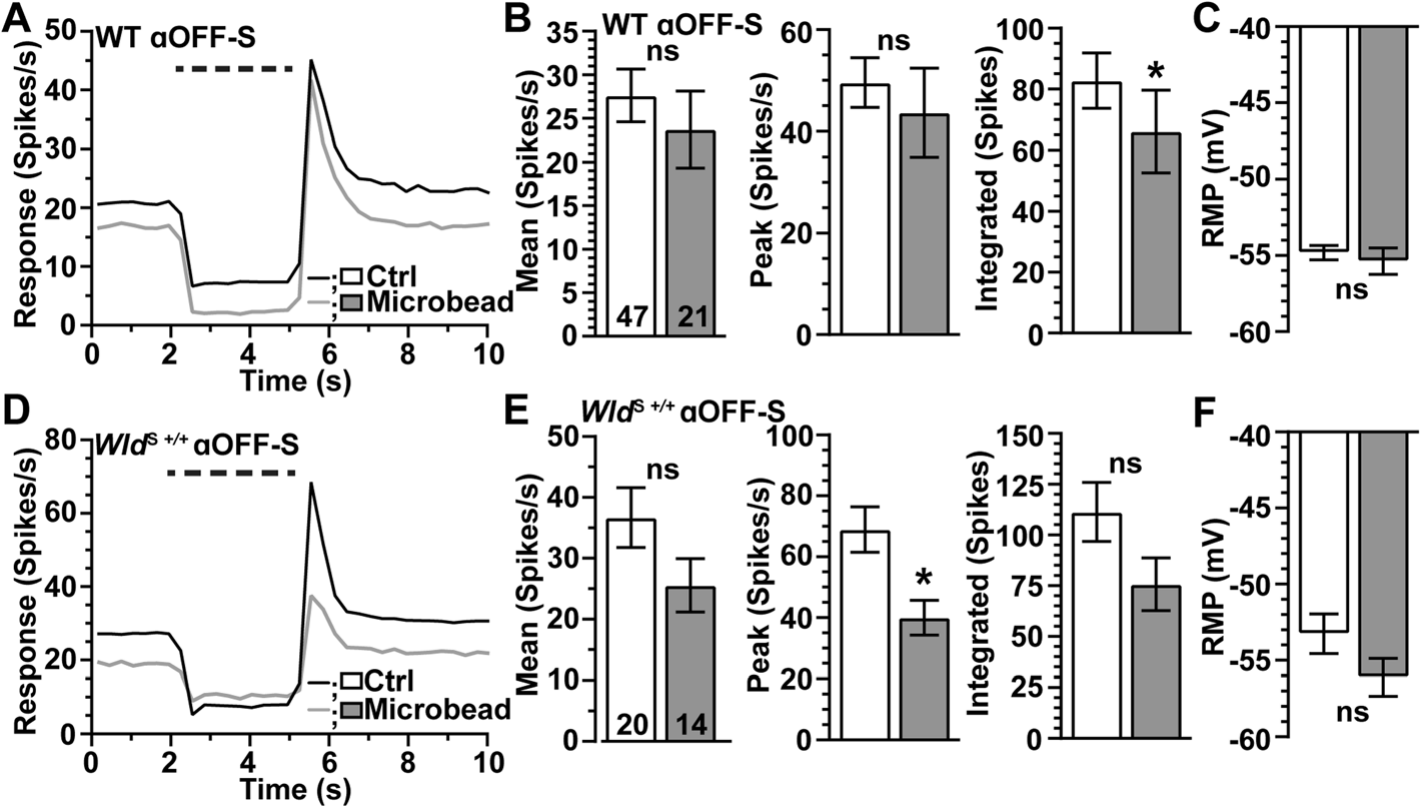
*Wld*^S+/+^ Does Not Protect Light-Evoked Responses of αOFF-S RGCs During IOP Elevation. **A** Average light-driven spike rate histograms (300 ms bin width) of WT αOFF-S RGCs from Ctrl and microbead eyes. **B** IOP elevation significantly reduced light-evoked integrated response of WT αOFF-S RGCs (− 20%, − 66.1 ± 13.5 vs. 82.8 ± 9 spikes, *p* = 0.011), **C** but RMP of αOFF-S RGCs from Ctrl and microbead-injected eyes is similar (*p* = 0.55). **D** Mean light-evoked spike rate histograms of *Wld*^S+/+^ αOFF-S RGCs from Ctrl and microbead eyes. **E** IOP elevation significantly decreased the peak response to light offset in *Wld*^S+/+^ αOFF-S RGCs (− 42%, 40 ± 5.7 vs. 69 ± 7.5 spikes/s, *p* = 0.01), and modestly reduced the mean (*p* = 0.12) and integrated (*p* = 0.09) response to light offset. **F** RMP is similar for *Wld*^*S*+/+^ αOFF-S RGCs from Ctrl and microbead-injected eyes (*p* = 0.13). Statistics: Student’s *t*-test **(B, C, E, F)**; Mann-Whitney test (**E**, peak spike rate). Data = mean ± SEM

With a month of elevated IOP, αOFF-S RGCs in WT retina demonstrated less overall activity before, during and after light presentation (Fig. 7A), consistent with our previous work [29]. As before, the integrated response to light offset reduced significantly (− 20%, *p* = 0.011), while the mean and peak response to offset did not change (*p* ≥ 0.12, Fig. 7B). The RMP did not change with elevated IOP, in contrast to the more depolarized RMP of WT αON-S RGCs (*p* = 0.55, Fig. 4C). In contrast to αON-S RGCs, elevated IOP decreased the light response of αOFF-S RGCs from *Wld*^S+/+^ retinas (Fig. 7D), reducing the peak firing rate by 42% (*p* = 0.01, Fig. 7E). Thus, *Wld*^S+/+^ is insufficient for the protection of mechanisms driving light-evoked responses of αOFF-S RGCs following a month of IOP elevation Table 1.

**Table 1:**
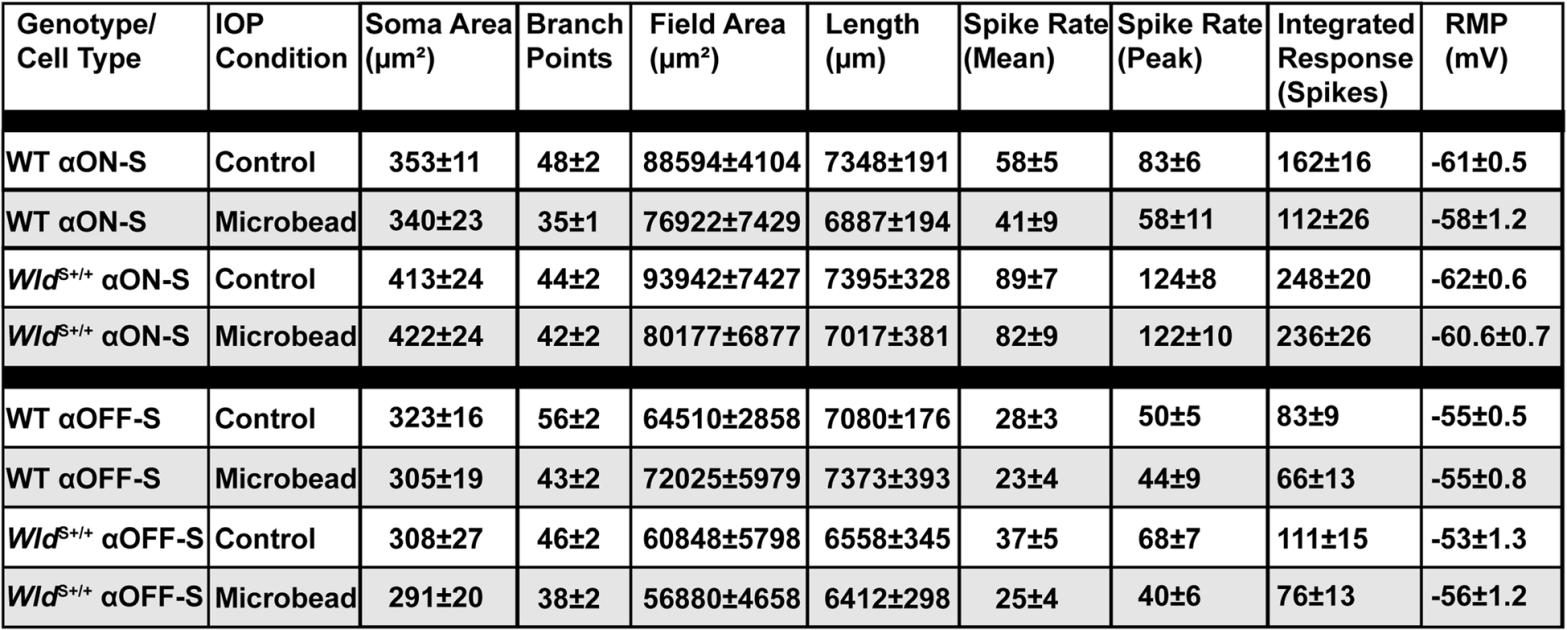
Descriptive Statistics for Dendritic Morphological and Physiological Measurements of αON- and αOFF-S RGCs from Control and Microbead Eyes of WT and *Wld*^S*+/+*^ Animals. Values are expressed as mean ± SEM

### Age-dependent influence of *Wld*^S+/+^ on axon transport and spatial acuity

**Fig. 8:**
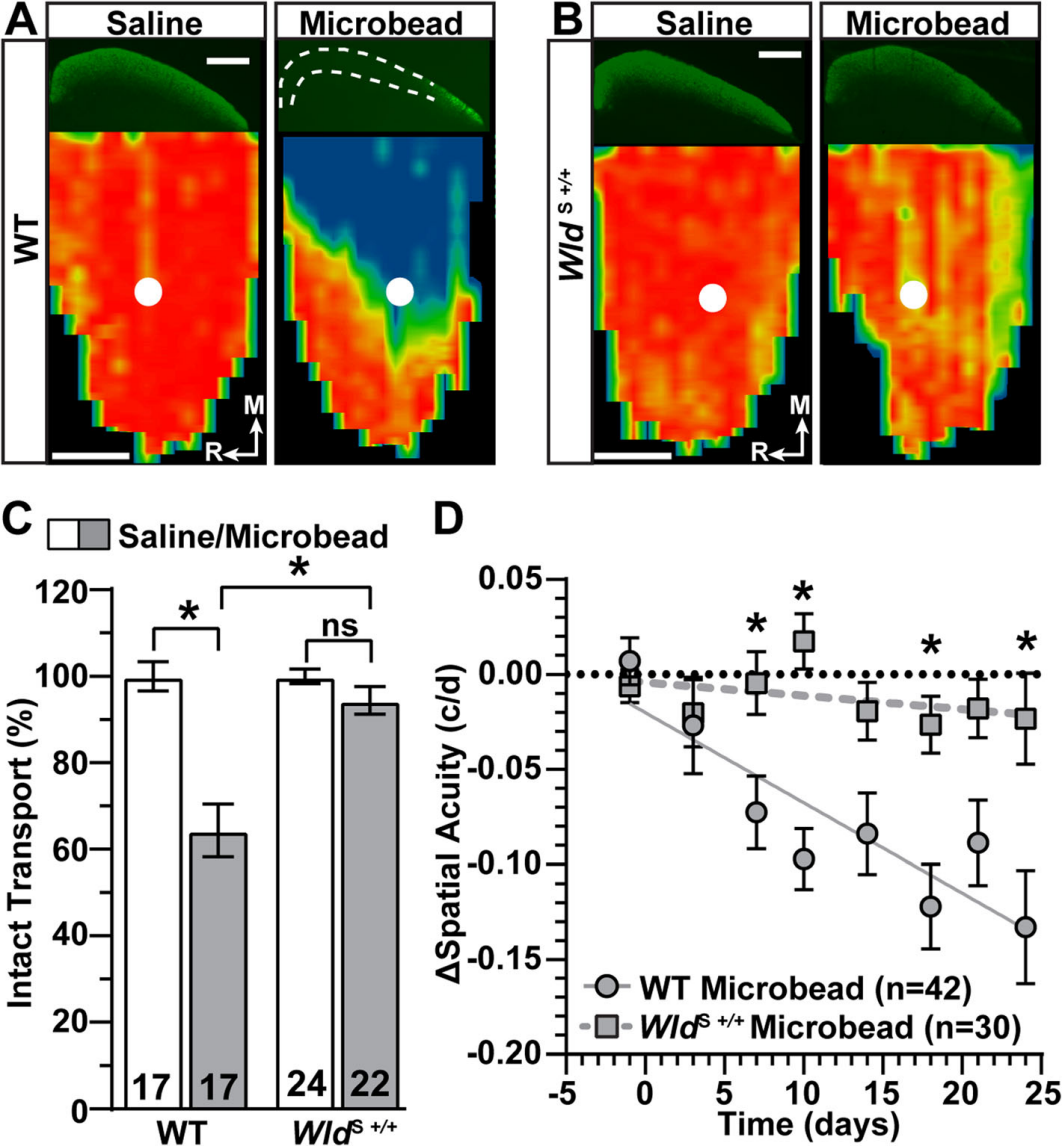
*Wld*^*S*+/+^ Protects Anterograde Axon Transport and Spatial Acuity Following IOP Elevation in Young Mice. **A, B** Representative coronal sections (top) of the superior colliculus (SC) following intravitreal injection of CTB-488 (green) into saline- and microbead-injected eyes of WT **(A)** and *Wld*^S*+/+*^ **(B)** mice. Transport deficits indicated by dashed lines. Retinotopic maps (bottom) reconstructed from serial sections of SC with optic disc indicated (white circles). Density of the transported CTB signal ranges from 0% (blue) to 50% (green) to 100% (red). Medial (M) and rostral (R) orientations are indicated. Scale bar = 500 μm. **C** Quantification of the intact anterograde transport (%) to the SC from saline- and microbead-injected eyes of WT and *Wld*^S*+/+*^ mice. IOP elevation significantly decreased transport in WT mice (*p* < 0.001) but transport is preserved in *Wld*^S+/+^ animals (*p* > 0.99) and significantly greater than WT microbead eyes (*p* = 0.007). **D** Difference in spatial acuity threshold (cycles/degree, c/d) between saline and microbead eyes (microbead-control, Δ) from WT and *Wld*^S*+/+*^ mice. Spatial acuity linearly diminishes over time in WT (R^2^ = 0.80, *p* = 0.002) but remains stable in *Wld*^S+/+^ mice (R^2^ = 0.18, *p* = 0.283). Δ spatial acuity is significantly greater in *Wld*^S+/+^ versus WT mice (*, *p* ≤ 0.05). Statistics: Kruskal-Wallis test, Dunn’s post hoc **(C)**, Linear mixed model **(D)**, Linear regression **(D)**. Data = mean ± SEM

The protective capacity of *Wld*^S^ depends on age, even though *Wld*^S^ expression does not change with age [20, 49, 50]. Here, we tested how aging impacts *Wld*^S+/+^ protection of anterograde axon transport to the superior colliculus (SC) and spatial frequency threshold (i.e., spatial acuity) in the context of glaucoma. We first established a basis for testing the influence of aging on *Wld*^S+/+^ protective capacity in young mice (aged 6–8 weeks). A month of IOP elevation significantly degraded anterograde transport of cholera toxin subunit B (CTB) to the SC in WT mice (Fig. 8A), while transport from *Wld*^S+/+^ eyes remained largely intact (Fig. 8B). When quantified, elevated IOP reduced the percentage of intact transport to the SC by 36% compared to control in WT animals (*p* < 0.001, Fig. 8C), a significant difference compared to *Wld*^S+/+^ (*p* = 0.007, Fig. 8C). For WT mice, spatial acuity significantly diminished over the experimental period (R^2^ = 0.80, *p* = 0.002), while acuity for *Wld*^S+/+^ did not change (R^2^ = 0.18, *p* = 0.28) and remained better than WT over the course of IOP elevation (*p* ≤ 0.05, Fig. 8D). Our results indicate *Wld*^S+/+^ prevents significant loss of axon transport and spatial acuity following 1 month of IOP elevation in young mice.

**Fig. 9:**
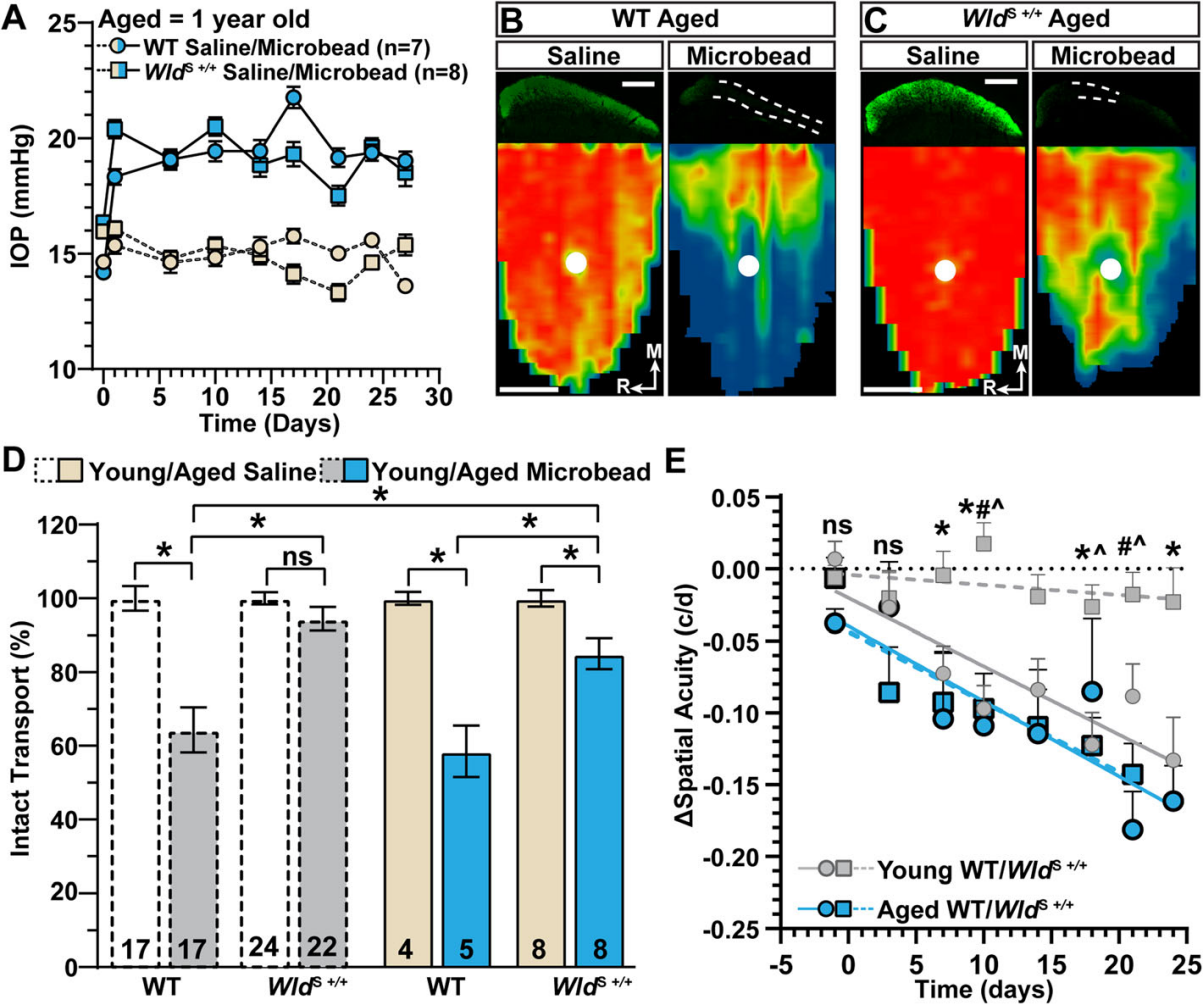
Aging Reduces the Neuroprotective Capacity of *Wld*^S+/+^. **A** IOP of aged WT (circles) and *Wld*^S*+/+*^ (squares) mice before (day 0) and after (days ≥1) a single unilateral injection of microbeads (blue symbols) or equivalent volume of saline (beige symbols). Microbeads elevated IOP by 29.5% in aged WT (19.45 ± 1.5 vs. 15.01 ± 1.3 mmHg, *p* < 0.001) and 29.7% in *Wld*^S*+/+*^ eyes compared to saline-injected eyes (19.23 ± 1.6 vs. 14.82 ± 1.3 mmHg, *p* < 0.001). **B, C** Example coronal sections (top) of the SC after intravitreal injection of CTB-488 (green) into saline- and microbead eyes of aged WT **(B)** and *Wld*^S*+/+*^ mice **(C)**. Transport deficits are indicated by dashed lines. Retinotopic maps (bottom) recreated from SC sections with optic disc indicated by white filled circles. Scale bar = 500 μm. **D** Quantification of anterograde axon transport to the SC. Data from young (1.5–2.5 months old) animals are replotted from Fig. 8C for comparison. We did not detect a difference in the percent of intact transport to the SCs corresponding to saline eyes of young vs. aged WT and *Wld*^S*+/+*^ animals (*p* = 0.60). For aged animals, IOP elevation decreased transport in WT (*p* < 0.001) and *Wld*^S*+/+*^ mice (*p* = 0.03) when compared to respective saline eyes. Axon transport of aged *Wld*^S*+/+*^ microbead-injected eyes was greater than that observed in SC of aged WT microbead eyes (*p* = 0.001). **E** Quantification of Δ spatial acuity. Data for young animals replotted from Fig. 8D for comparison. Spatial acuity linearly diminishes over the duration of IOP elevation in aged WT (R^2^ = 0.74, *p* = 0.0064) and *Wld*^S+/+^ mice (R^2^ = 0.81, *p* = 0.0058). Statistics: Student’s *t*-test **(A)**, Kruskal-Wallis test, Dunn’s post hoc **(D)** Linear mixed model **(E)**, Linear regression **(E)**. Multiple comparison significance indicators: * Young WT vs. Young *Wld*^S+/+^; ^**#**^ Aged WT vs. Young *Wld*^S+/+^; **^** Young *Wld*^S+/+^ vs. Aged *Wld*^S+/+^ **(E)**. Data presented = mean ± SEM

Next, we tested the influence of age on the protective capacity of *Wld*^S^ on anterograde axon transport and spatial acuity following IOP elevation. We again elevated IOP by unilateral microbead injection but using aged (1 year old) WT and *Wld*^S+/+^ animals (Fig. 9A). A single injection of microbeads similarly increased IOP in both WT (+ 29.6%, 19.45 ± 1.52 vs. 15.01 ± 1.28 mmHg) and *Wld*^S^ eyes (+ 29.76, 19.23 ± 1.67 vs. 14.82 ± 1.37 mmHg). Four weeks of IOP elevation reduced intact anterograde axon transport in aged WT mice slightly more than it did for young, 41% vs. 36% (*p* = 0.74; Fig. 9B, D). Interestingly, aged *Wld*^S+/+^ animals also demonstrated degraded transport with elevated IOP compared to control eyes (− 15%; *p* ≤ 0.03, Fig. 9C,D), though still retaining significantly more intact transport compared to either young or aged WT eyes challenged by elevated IOP (*p* ≤ 0.002). Aging also influenced spatial acuity. Elevated IOP induced a decrease not only in WT (*p* = 0.0064), but also in *Wld*^S+/+^ animals (*p* = 0.0058, Fig. 9E), which significantly exceeded the rate of reduction in younger *Wld*^S+/+^ animals (*p* < 0.001). The rate of degradation in acuity was similar between aged WT and *Wld*^S+/+^ mice (*p* = 0.35). Thus, aging reduces the neuroprotective benefit of *Wld*^S+/+^ not only on axonal transport but also throughout the optic projection.

## Discussion

### Extent of neuroprotection by *Wld*^S^ is dependent on RGC type during glaucoma

Our first finding is neuroprotection by *Wld*^S+/+^ during glaucoma appears to depend on RGC type. Our results suggest *Wld*^S+/+^ confers neuroprotection in αON-S RGCs but not αOFF-S RGCs. Following 1 month of IOP elevation by microbead occlusion, WT αON-S RGC dendritic crossings (Sholl analysis) and branch points decreased while *Wld*^S+/+^ αON-S RGC dendritic arbors remained intact (Fig. 3). This result suggests *Wld*^*S*+/+^ not only protects RGC somas and axons during glaucoma [22], but extends to dendrites of RGCs [25]. Regarding RGC physiology, we find 1 month of elevated IOP depolarizes RMP and reduces the mean, peak, and integrated response to light of WT αON-S RGCs (Fig. 4A-C). While short term elevations in IOP increase RGC light responses and excitability, prolonged IOP elevation diminishes both [29, 51]. For *Wld*^S+/+^ αON-S RGCs, RMP and light response properties are unaffected by IOP elevation (Fig. 4D-F). Our finding substantiates evidence that *Wld*^S+/+^ preserves the RGC pattern electroretinogram response during DBA2/J glaucoma [22]. Furthermore, our data suggests *Wld*^S+/+^ either indirectly or directly protects mechanisms that generate RMP and voltage-gated responses of αON-S RGCs [29, 51].

Although *Wld*^S+/+^ provides extensive protection during glaucoma for dendritic arbors and mechanisms that drive RMP and the light-evoked response of αON-S RGCs, neuroprotection by *Wld*^S+/+^ is not robustly afforded to αOFF-S RGCs. We find IOP elevation significantly reduces the number of dendritic branch points of αOFF-S RGCs from WT and *Wld*^S+/+^ eyes (Fig. 6C, F). Moreover, we find 1 month of IOP elevation decreases the integrated light-evoked response of WT αOFF-S RGCs (Fig. 7B) and the peak response of *Wld*^S+/+^ αOFF-S RGCs (Fig. 7E).

Based on these data, *Wld*^S+/+^ appears to protect αON-S RGCs but not αOFF-S cells. In support for this argument, evidence suggests OFF RGCs are more vulnerable to stress caused by IOP elevation [31–33]. Although this is a tantalizing conclusion, qualitative analysis reveals *Wld*^S^ protein is expressed in the nuclei of most cells in the RGC layer of *Wld*^S+/+^ mice, indicating RGCs should be equally protected from stress by *Wld*^S^ [52]. However, the magnitude of neuroprotection following acute axonal injury is correlated with *Wld*^S^ expression levels in α- and γ-motor neuron neurons [53]. Thus, the possibility exists that *Wld*^S^ expression is dependent on RGC type.

The discrepancy between the extent of neuroprotection provided by *Wld*^S^ to αON- and αOFF-S RGCs is further highlighted by the fact the duration of *Wld*^S^ neuroprotection is dependent on glaucoma model. *Wld*^S^ neuroprotection lasts months in chronic DBA2/J glaucoma but is restricted to 2–4 weeks following IOP elevation by photocoagulation of the trabecular meshwork [22, 52]. Therefore, it might be the case that both αON- and αOFF-S RGC respond to *Wld*^S^ during stress, but the duration of protection by *Wld*^S^ is more transient for αOFF-S RGCs.

The duration of neuroprotection by *Wld*^S^ may be shorter for αOFF-S RGCs due to their intrinsic physiological properties. αOFF-S RGCs intrinsically generate greater spontaneous activity compared to αON-S RGCs (Figs. 2 and 5B). Thus, αOFF-S RGCs may be more sensitive to stress due to the metabolic burden of persistent action potential generation and maintaining RMP. Cell excitability determines the magnitude of neuroprotection by *Wld*^S^ after nerve sectioning. “Enhancing axon excitability by high-frequency stimulation or blunting excitability by blocking voltage-gated sodium channels with tetrodotoxin both accelerate axon degeneration following axotomy of neuromuscular junctions in *Wld*^S^ mice” [54]. Considering these findings, αOFF-S RGCs may be more vulnerable to stress due to their intrinsically high spontaneous activity, which is further enhanced during early progression of glaucoma [29, 34].

### Neuroprotective capacity of *Wld*^S^ is dependent on age during glaucoma

Our second key finding is neuroprotection by *Wld*^S^ on anterograde axonal transport and spatial acuity during glaucoma is dependent on age. As we have previously shown [29, 43], IOP elevation significantly reduces anterograde axonal transport and spatial acuity in young adult WT mice (Fig. 8). Both anterograde axon transport and spatial acuity are protected during glaucoma in young adult *Wld*^S+/+^ mice (Fig. 8). Our data provide additional confirmatory evidence that *Wld*^S+/+^ provides neuroprotection for axon transport during glaucoma [22–24]. Furthermore, our results indicate if axonopathy caused by IOP elevation is reduced, as is the case for young adult *Wld*^S+/+^ animals (Fig. 8C), degradation of spatial acuity slows (Fig. 8D).

Although we find anterograde axonal transport and spatial acuity are preserved in young adult *Wld*^S+/+^ animals, neuroprotection by *Wld*^S^ is reduced in aged, one-year old, animals following IOP elevation (Fig. 9). Even so, *Wld*^S^ still offers some protection on anterograde axon transport in aged mice relative to age-matched WTs (Fig. 9B-D). However, IOP elevation significantly reduces spatial acuity similarly for aged WT and *Wld*^S+/+^ mice (Fig. 9E).

Our finding that neuroprotection by *Wld*^S+/+^ depends on age is surprising because axon integrity and RGC pattern electroretinogram responses are preserved in 10 to 12 month old DBA2/J.Wld^S^ mice [22]. However, when the age range is narrowed to include only 12 month old DBA2/J.Wld^S^ mice, optic nerves possess more degenerating axons compared to age-matched DBA2/J.Wld^S^ mice supplemented with nicotinamide, indicating neuroprotection by *Wld*^S^ on axonopathy is reduced by advanced age in chronic DBA2/J.Wld^S^ glaucoma [24]. These data suggest neuroprotection by *Wld*^S^ falls off precipitously between 10 to 12 months of age in DBA2/J glaucoma. Following an inducible injury, the neuroprotective capacity of *Wld*^S^ is also age dependent, even though *Wld*^S^ expression levels do not change with age [20, 49, 50]. Taken together, the degree of neuroprotection by *Wld*^S^ appears to be highly sensitive to age, limiting the utility of targeting only *Wld*^S^ to block the progression of age-related neurodegenerative diseases [24, 55, 56].

## Conclusions

We have demonstrated that *Wld*^S+/+^ does not equally protect all RGC types even in young adult animals during glaucoma and neuroprotection by *Wld*^S+/+^ on axonopathy is dependent on age. We find αOFF-S RGCs from *Wld*^S+/+^ retinas are similarly susceptible to dendritic degeneration and degradation of RGC signaling during glaucoma as their WT counterparts. While *Wld*^S+/+^ protects αON-S RGC dendrites and light-evoked activity. The influence of *Wld*^S+/+^ on anterograde axonal transport and spatial acuity is significantly blunted in aged animals. Although *Wld*^S+/+^ confers significant neuroprotection of anterograde axon transport and spatial acuity during axonopathy in otherwise uncompromised systems, this protection is limited at the level of different RGC types and is age dependent.

## Data Availability

The datasets used and/or analyzed during the current study are available from the corresponding author on reasonable request.

## Abbreviations

RGC: Retinal ganglion cells
*Wld*^S^: *Slow Wallerian degeneration*
IOP: Intraocular pressure
WT: Wild type
RBPMS: RNA-binding protein with multiple splicing
RMP: Resting membrane potential
CTB: Cholera toxin subunit B
IPL: Inner plexiform layer
ChAT: Choline acetyltransferase
SC: Superior colliculus
